# Supplementary material for: Genomic Surveillance of Rabies Virus in Georgian Canines
Source: Viruses. 2023 Aug 24;15(9):1797. doi: 10.3390/v15091797 (PMC10537093; doi:10.3390/v15091797)
Supplement: Supplementary file 1 [file viruses-15-01797-s001.zip › Supplementary Figures S1-S4-BCS edits.pdf]

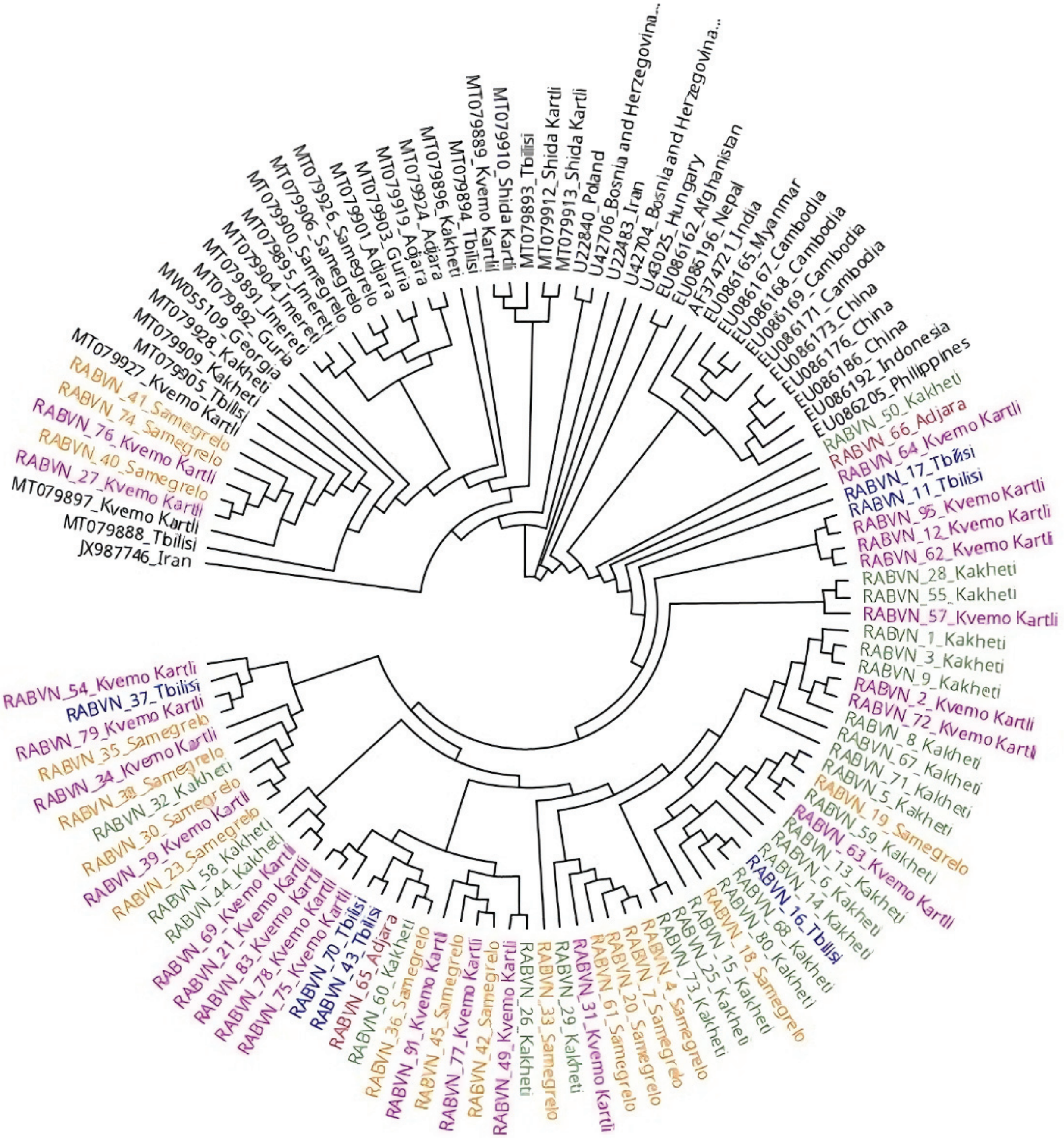

Supplemenatary Figure S1. Phylogenetic tree of Georgian lyssavirus *N* gene PCR products. Sanger sequences of PCR products of the N-terminus (approximately 542 bp) of lyssavirus *N* genes of RABV-GEO isolates were subjected to phylogenetic relatedness analysis using Geneious Prime software (Dotmatics, Boston, MA).

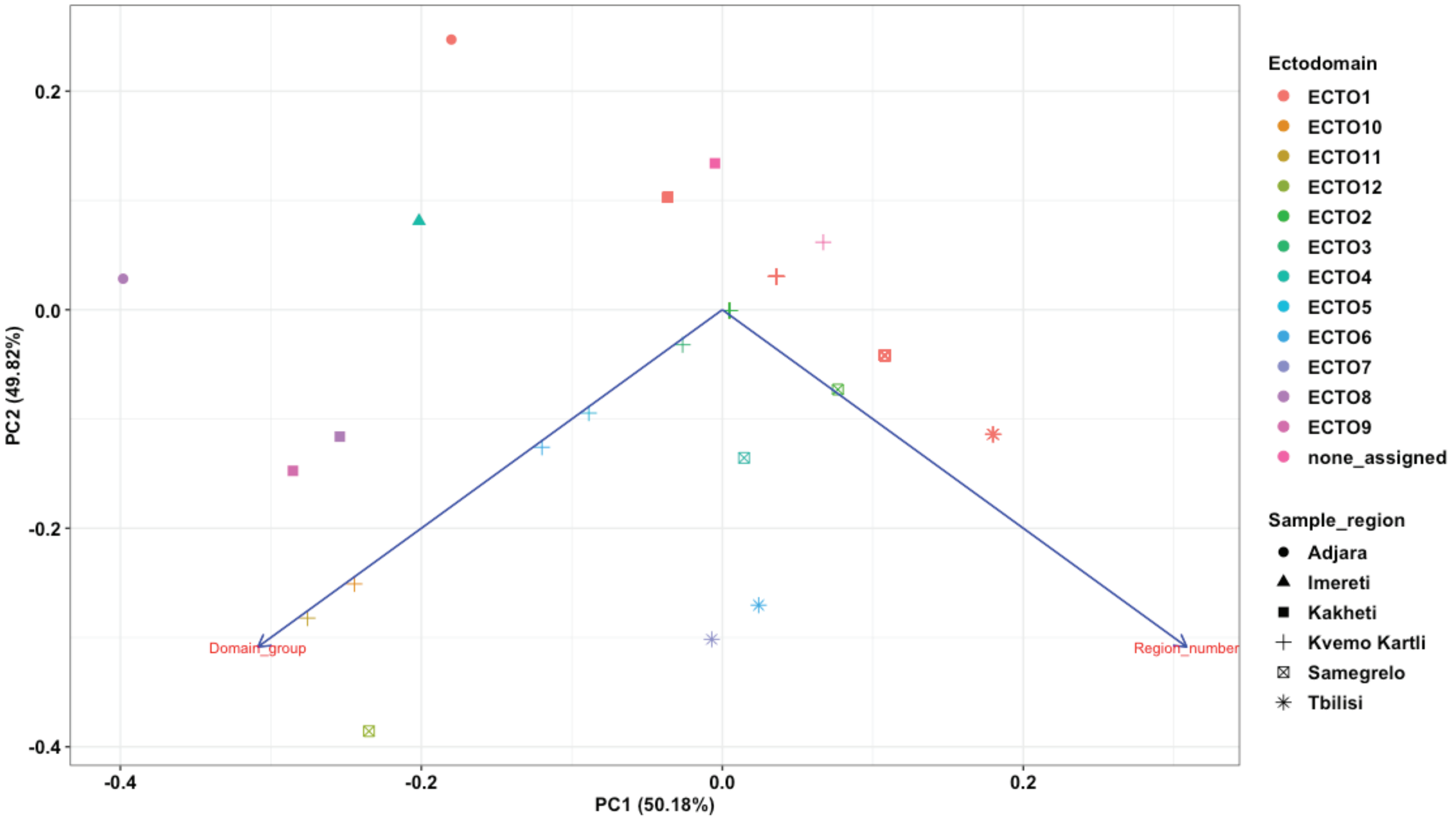

Supplementary Figure S2. PCA of ectodomain group and subregion. Principal components analysis of ectodomain groups and subregions of Georgia from which samples were collected. Points indicate score for principal components 1 and 2 and are color coded in correspondence with ectodomain group assignment and shaped by geographical origin of the sample. Vectors (blue arrows) show the loadings, indicating the weight each variable has on the principal component. Due to a vector angle close to 90°, we interpret these variables as unlikely to be correlated.

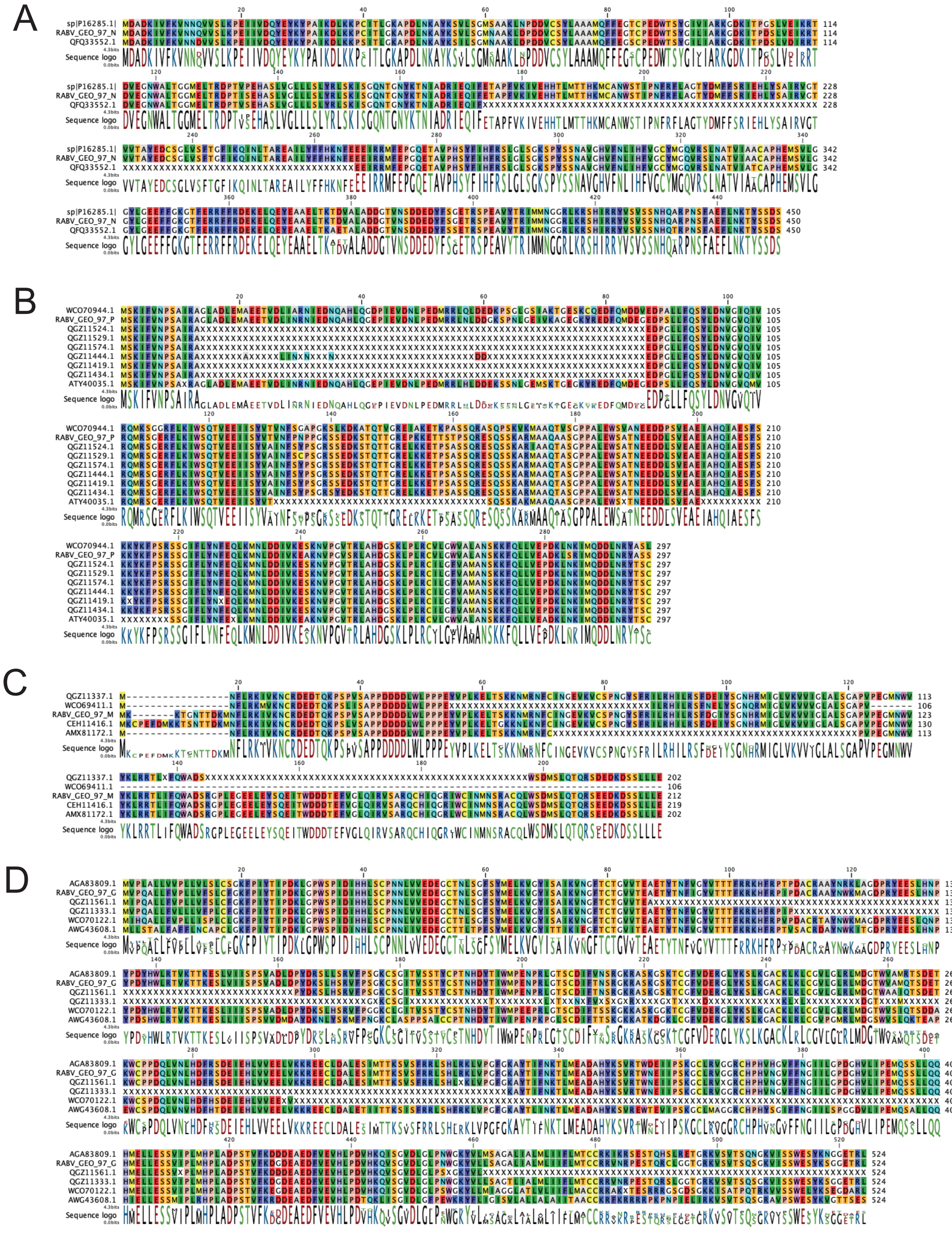

Supplementary Figure S3. Alignment of representative amino acid sequences from RABV protein clusters. (A) N protein. (B) P protein. (C) M protein (D) G protein.

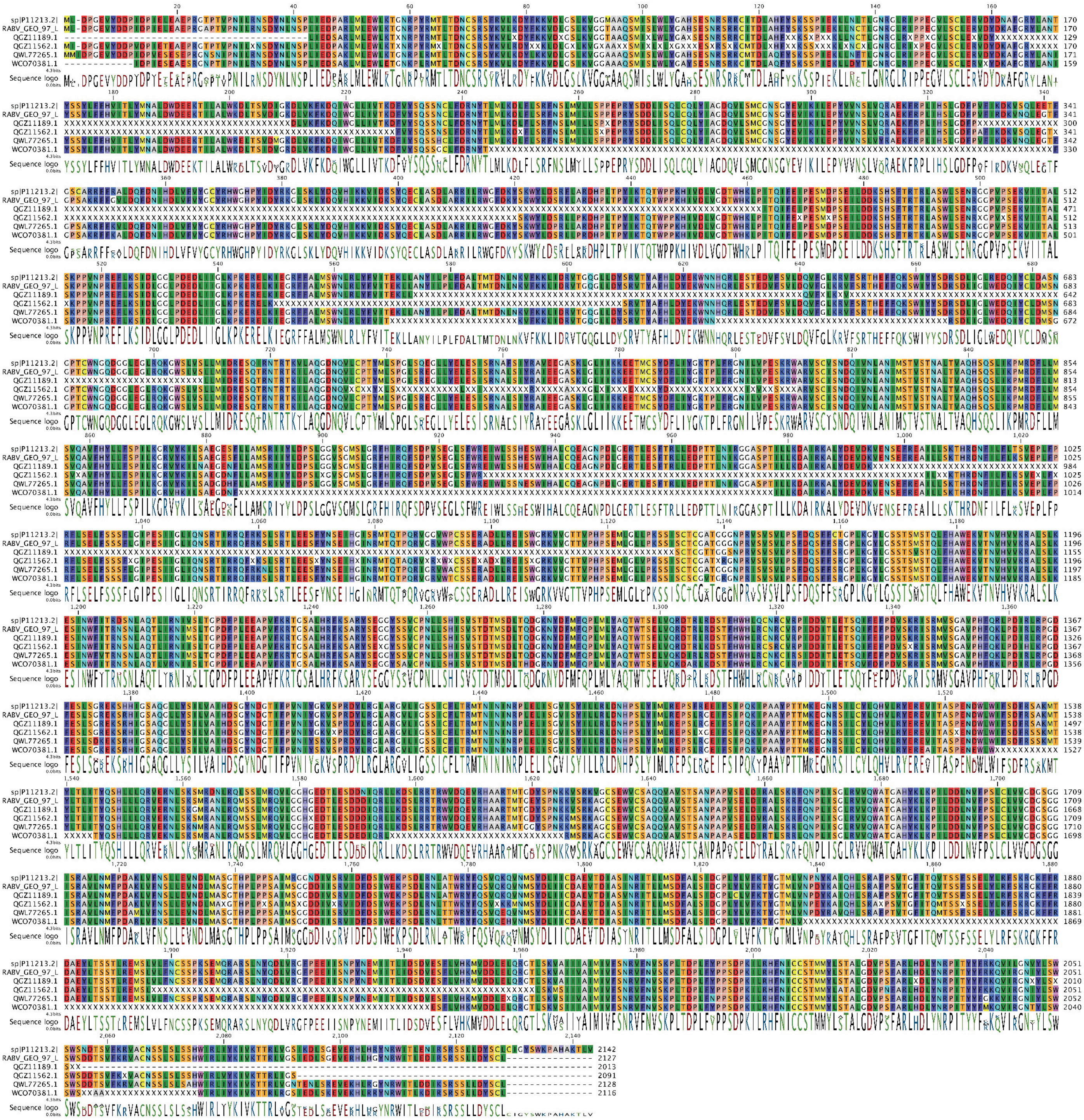

Supplementary Figure S4. Alignment of representative amino acid sequences from the RABV L protein cluster.
