# Supplementary material for: Genomic Surveillance of Rabies Virus in Georgian Canines
Source: Viruses. 2023 Aug 24;15(9):1797. doi: 10.3390/v15091797 (PMC10537093; doi:10.3390/v15091797)
Supplement: Supplementary file 1 [file viruses-15-01797-s001.zip › Supplementary_tables-BCS confirmed.pdf]

Supplementary Table S1

Sample name	Host Species	RT-PCR RABV	RABV sequencing reads	Depth of Coverage	Breadth of Coverage	GenBank Sequence ID	Clade	Ectodomain Group	Location of sample collection	Date of sample collection
RABV-GEO-1	Dog	+	0.13%	703	CC	OQ603614	Cosmopolitan	ECTO1	Kakheti	1/15/2018
RABV-GEO-2	Dog	+	0.20%	2,000	CC	OQ603624	Cosmopolitan	ECTO2	Kvemo Kartli	1/23/2018
RABV-GEO-3	Dog	+	0.15%	1,207	CC	OQ603632	Cosmopolitan	ECTO1	Kakheti	1/29/2018
RABV-GEO-4	Dog	+	0.10%	673	CC	OQ603643	Cosmopolitan	ECTO1	Samegrelo	2/6/2018
RABV-GEO-5	Dog	+	0.16%	1,426	CC	OQ603657	Cosmopolitan	ECTO1	Kakheti	2/12/2018
RABV-GEO-6	Dog	+	0.19%	57	CC	OQ603609	Cosmopolitan	ECTO1	Kakheti	3/19/2018
RABV-GEO-7	Dog	+	0.04%	80	CC	OQ603668	Cosmopolitan	ECTO1	Samegrelo	4/18/2018
RABV-GEO-8	Dog	+	0.14%	930	CC	OQ603679	Cosmopolitan	ECTO1	Kakheti	4/26/2018
RABV-GEO-9	Dog	+	0.23%	164	CC	OQ603612	Cosmopolitan	ECTO1	Kakheti	4/27/2018
RABV-GEO-10	Dog	+	0.14%	2,878	CC	OQ603615	Cosmopolitan	ECTO1	Samegrelo	5/10/2018
RABV-GEO-11	Dog	+	0.17%	1,056	CC	OQ603616	Cosmopolitan	ECTO1	Tbilisi	5/14/2018
RABV-GEO-12	Dog	+	0.13%	783	CC	OQ603617	Cosmopolitan	ECTO3	Kvemo Kartli	5/16/2018
RABV-GEO-13	Dog	+	0.03%	20,480	CC	OQ603610	Cosmopolitan	ECTO1	Kakheti	6/4/2018
RABV-GEO-14	Dog	+	0.26%	1,864	CC	OQ603618	Cosmopolitan	ECTO1	Kakheti	7/2/2018
RABV-GEO-15	Dog	+	0.10%	431	CC	OQ603619	Cosmopolitan	ECTO1	Kakheti	7/5/2018
RABV-GEO-16	Dog	+	0.93%	12,251	CC	OQ603620	Cosmopolitan	ECTO1	Tbilisi	8/7/2018
RABV-GEO-17	Dog	+	0.63%	11,796	CC	OQ603621	Cosmopolitan	ECTO1	Tbilisi	8/9/2018
RABV-GEO-18	Dog	+	0.39%	2,869	CC	OQ603622	Cosmopolitan	ECTO1	Samegrelo	8/20/2018
RABV-GEO-19	Dog	+	0.16%	945	NCC	OQ603623	Cosmopolitan	ECTO4	Samegrelo	9/27/2018
RABV-GEO-20	Dog	+	0.40%	852	CC	OQ603611	Cosmopolitan	ECTO12	Samegrelo	10/23/2018
RABV-GEO-21	Dog	+	0.15%	877	CC	OQ603625	Cosmopolitan	ECTO1	Kvemo Kartli	01/14/2020
RABV-GEO-23	Dog	+	0.04%	58	CC	OQ603626	Cosmopolitan	ECTO1	Samegrelo	01/22/2019
RABV-GEO-25	Dog	+	0.19%	1,650	CC	OQ603627	Cosmopolitan	ECTO1	Kakheti	01/28/2019
RABV-GEO-26	Dog	+	0.11%	1,238	CC	OQ603628	Cosmopolitan	ECTO1	Kakheti	02/13/2020
RABV-GEO-27	Dog	+	0.12%	723	CC	OQ603629	Cosmopolitan	ECTO5	Kvemo Kartli	02/18/2019
RABV-GEO-28	Dog	+	0.12%	1,879	CC	OQ603630	Cosmopolitan	ECTO1	Kakheti	02/21/2020
RABV-GEO-29	Dog	+	0.17%	827	CC	OQ603631	Cosmopolitan	ECTO1	Kakheti	03/04/2019
RABV-GEO-30	Dog	+	0.08%	297	CC	OQ603633	Cosmopolitan	ECTO1	Samegrelo	02/28/2020
RABV-GEO-31	Dog	+	0.13%	837	CC	OQ603634	Cosmopolitan	ECTO2	Kvemo Kartli	04/02/2019
RABV-GEO-32	Dog	+	0.19%	1,933	CC	OQ603635	Cosmopolitan	ECTO1	Kakheti	04/11/2019
RABV-GEO-33	Dog	+	0.06%	218	CC	OQ603636	Cosmopolitan	ECTO1	Samegrelo	04/15/2019
RABV-GEO-34	Dog	+	0.09%	731	CC	OQ603637	Cosmopolitan	ECTO1	Kvemo Kartli	04/22/2019
RABV-GEO-35	Dog	+	0.16%	2,310	CC	OQ603638	Cosmopolitan	ECTO1	Samegrelo	04/24/2019
RABV-GEO-36	Dog	+	0.05%	375	CC	OQ603639	Cosmopolitan	ECTO1	Samegrelo	05/15/2019
RABV-GEO-37	Dog	+	0.09%	2,061	CC	OQ603640	Cosmopolitan	ECTO6	Tbilisi	05/20/2019
RABV-GEO-38	Dog	+	0.17%	3,427	CC	OQ603641	Cosmopolitan	ECTO2	Samegrelo	05/23/2019
RABV-GEO-39	Dog	+	0.21%	4,533	CC	OQ603642	Cosmopolitan	ECTO2	Kvemo Kartli	05/30/2019
RABV-GEO-40	Dog	+	0.05%	210	CC	OQ603644	Cosmopolitan	ECTO1	Samegrelo	06/03/2019
RABV-GEO-41	Dog	+	0.09%	856	CC	OQ603645	Cosmopolitan	ECTO1	Samegrelo	06/26/2019
RABV-GEO-42	Dog	+	0.04%	188	CC	OQ603646	Cosmopolitan	ECTO1	Samegrelo	06/26/2019
RABV-GEO-43	Dog	+	0.07%	441	CC	OQ603647	Cosmopolitan	ECTO7	Tbilisi	06/28/2019

RABV-GEO-44	Dog	+	0.09%	717	CC	OQ603648	Cosmopolitan	ECTO1	Kakheti	07/01/2019
RABV-GEO-45	Dog	+	0.09%	801	CC	OQ603649	Cosmopolitan	ECTO1	Samegrelo	07/05/2019
RABV-GEO-49	Dog	+	0.19%	3,886	CC	OQ603650	Cosmopolitan	ECTO1	Kvemo Kartli	08/30/2019
RABV-GEO-50	Dog	+	0.15%	2,040	CC	OQ603651	Cosmopolitan	ECTO1	Kakheti	09/25/2019
RABV-GEO-54	Dog	+	0.05%	459	CC	OQ603652	Cosmopolitan	ECTO6	Kvemo Kartli	12/25/2019
RABV-GEO-55	Dog	+	0.07%	1,448	CC	OQ603653	Cosmopolitan	ECTO1	Kakheti	12/26/2019
RABV-GEO-57	Dog	+	0.09%	3,303	CC	OQ603654	Cosmopolitan	ECTO1	Kvemo Kartli	12/30/2019
RABV-GEO-58	Dog	+	0.11%	2,247	CC	OQ603655	Cosmopolitan	ECTO1	Kakheti	11/21/2018
RABV-GEO-59	Dog	+	0.16%	1,695	CC	OQ603656	Cosmopolitan	ECTO1	Kakheti	11/27/2018
RABV-GEO-60	Dog	+	0.16%	4,158	CC	OQ603658	Cosmopolitan	ECTO1	Kakheti	12/20/2018
RABV-GEO-61	Jackal	+	0.05%	244	CC	OQ603659	Cosmopolitan	ECTO1	Samegrelo	1/13/2020
RABV-GEO-62	Dog	+	0.13%	1,536	CC	OQ603660	Cosmopolitan	ECTO1	Kvemo Kartli	2/1/2021
RABV-GEO-63	Jackal	+	0.28%	5,656	CC	OQ603661	Cosmopolitan	ECTO1	Kvemo Kartli	2/1/2021
RABV-GEO-64	Dog	+	0.15%	720	CC	OQ603662	Cosmopolitan	ECTO1	Kvemo Kartli	2/3/2021
RABV-GEO-65	Dog	+	0.21%	1,167	CC	OQ603663	Cosmopolitan	ECTO1	Adjara	2/5/2021
RABV-GEO-66	Jackal	+	0.09%	289	CC	OQ603664	Cosmopolitan	ECTO8	Adjara	2/8/2021
RABV-GEO-67	Jackal	+	0.06%	17	CC	OQ603665	Cosmopolitan	ECTO8	Kakheti	2/15/2018
RABV-GEO-68	Dog	+	0.18%	1,159	CC	OQ603666	Cosmopolitan	ECTO1	Kakheti	3/1/2021
RABV-GEO-69	Dog	+	0.23%	943	CC	OQ603667	Cosmopolitan	ECTO1	Kvemo Kartli	2/28/2020
RABV-GEO-70	Dog	+	0.15%	399	CC	OQ603669	Cosmopolitan	ECTO1	Tbilisi	3/6/2020
RABV-GEO-71	Dog	+	0.44%	2,507	CC	OQ603670	Cosmopolitan	ECTO1	Kakheti	3/20/2020
RABV-GEO-72	Dog	+	0.54%	1,534	CC	OQ603671	Cosmopolitan	ECTO2	Kvemo Kartli	3/19/2020
RABV-GEO-73	Dog	+	0.25%	926	CC	OQ603672	Cosmopolitan	ECTO9	Kakheti	4/5/2021
RABV-GEO-74	Dog	+	0.12%	302	CC	OQ603673	Cosmopolitan	ECTO1	Samegrelo	3/24/2020
RABV-GEO-75	Dog	+	0.15%	532	CC	OQ603674	Cosmopolitan	ECTO10	Kvemo Kartli	3/26/2020
RABV-GEO-76	Dog	+	0.20%	1,182	CC	OQ603675	Cosmopolitan	ECTO1	Kvemo Kartli	4/7/2021
RABV-GEO-77	Dog	+	0.09%	270	CC	OQ603676	Cosmopolitan	ECTO11	Kvemo Kartli	4/8/2021
RABV-GEO-78	Dog	+	0.07%	138	CC	OQ603677	Cosmopolitan	ECTO1	Kvemo Kartli	4/7/2020
RABV-GEO-79	Dog	+	0.14%	360	CC	OQ603678	Cosmopolitan	ECTO1	Kvemo Kartli	4/8/2020
RABV-GEO-80	Jackal	+	0.35%	2,440	CC	OQ603680	Cosmopolitan	ECTO1	Kakheti	4/27/2020
RABV-GEO-83	Jackal	+	0.11%	39	CC	OQ603681	Cosmopolitan	ECTO1	Kvemo Kartli	6/8/2020
RABV-GEO-88	Jackal	-	0.17%	207	CC	OQ603682	Cosmopolitan	ECTO1	Kakheti	7/6/2021
RABV-GEO-91	Dog	+	0.10%	77	CC	OQ603683	Cosmopolitan	ECTO1	Kvemo Kartli	8/31/2020
RABV-GEO-95	Jackal	+	0.07%	6	NCC	OQ603684	Cosmopolitan	N/A	Kvemo Kartli	9/4/2019
RABV-GEO-96	Dog	+	0.06%	13	NCC	OQ603685	Cosmopolitan	ECTO1	Tbilisi	10/19/2020
RABV-GEO-97	Jackal	-	0.14%	46	CC	OQ603613	Cosmopolitan	ECTO4	Imereti	11/2/2021
RABV-GEO-100	Jackal	-	0.12%	3	N/A	N/A	ND	N/A	Kakheti	11/23/2018

Supplementary Table S2

Sample	Reference Position	Type	Length	Reference	Allele	Zygosity	Count	Coverage	Frequency	Forward/reverse balance	Average quality
RABV_19	86	SNV	1	A	G	Heterozygous	1562	3014	51.82	0.314	36.54545
RABV_19	92	SNV	1	C	A	Heterozygous	1666	3197	52.11	0.318	36.60744
RABV_19	330	SNV	1	N	C	Heterozygous	1713	4301	39.83	0.49	36.60304
RABV_19	330	SNV	1	N	T	Heterozygous	2574	4301	59.85	0.48	36.68298
RABV_19	641	SNV	1	T	C	Heterozygous	1068	3040	35.13	0.464	36.25655
RABV_19	836	SNV	1	A	G	Heterozygous	2319	3700	62.68	0.484	35.40535
RABV_19	914	SNV	1	C	T	Heterozygous	1472	3401	43.28	0.493	36.78261
RABV_19	1106	SNV	1	A	G	Heterozygous	1410	3700	38.11	0.428	36.52766
RABV_19	1148	SNV	1	A	G	Heterozygous	1464	3407	42.97	0.448	36.43579
RABV_19	1343	SNV	1	A	G	Heterozygous	1357	3072	44.17	0.352	36.53132
RABV_19	1397	SNV	1	A	G	Heterozygous	1237	2337	52.93	0.446	36.19968
RABV_19	1690	SNV	1	A	G	Heterozygous	953	2423	39.33	0.454	36.52361
RABV_19	1916	SNV	1	A	G	Heterozygous	1011	2081	48.58	0.495	36.28981
RABV_19	2147	SNV	1	N	C	Heterozygous	604	1166	51.8	0.474	36.11258
RABV_19	2147	SNV	1	N	T	Heterozygous	521	1166	44.68	0.441	36.42418
RABV_19	2267	SNV	1	A	G	Heterozygous	1755	3161	55.52	0.405	36.5396
RABV_19	2288	SNV	1	T	C	Heterozygous	2077	3543	58.62	0.387	36.66009
RABV_19	2386	SNV	1	T	C	Heterozygous	678	1933	35.08	0.302	35.73451
RABV_19	2799	SNV	1	A	C	Heterozygous	1348	2528	53.32	0.452	36.47033
RABV_19	3097	SNV	1	C	G	Heterozygous	972	1953	49.77	0.325	36.31687
RABV_19	5307	SNV	1	C	T	Heterozygous	912	1732	52.66	0.409	36.48684
RABV_19	7170	SNV	1	N	A	Heterozygous	648	1256	51.59	0.478	36.47531
RABV_19	7170	SNV	1	N	G	Heterozygous	590	1256	46.97	0.459	35.89153
RABV_19	9093	SNV	1	T	G	Heterozygous	1273	2922	43.57	0.5	35.95994
RABV_19	9429	SNV	1	T	G	Heterozygous	1374	2401	57.23	0.486	36.70306
RABV_19	10707	SNV	1	A	G	Heterozygous	1074	2648	40.56	0.443	36.63687
RABV_19	11893	SNV	1	A	T	Heterozygous	12	28	42.86	0.5	37
RABV_19	1411	SNV	1	A	G	Homozygous	1202	2144	56.06	0.433	36.68552
RABV_19	1514	SNV	1	A	G	Homozygous	1001	1745	57.36	0.45	36.46853
RABV_19	3430	SNV	1	C	T	Homozygous	2172	2883	75.34	0.458	36.4116
RABV_19	3457	SNV	1	A	C	Homozygous	2457	3173	77.43	0.437	36.3195
RABV_19	3572	SNV	1	A	G	Homozygous	1888	2622	72.01	0.438	36.30085
RABV_19	3646	SNV	1	T	C	Homozygous	1585	2343	67.65	0.415	36.53438
RABV_19	3700	SNV	1	A	G	Homozygous	1807	2618	69.02	0.49	36.16989
RABV_19	3730	SNV	1	T	C	Homozygous	2137	3005	71.11	0.464	36.22789
RABV_19	3817	SNV	1	C	T	Homozygous	1766	2505	70.5	0.465	36.67497
RABV_19	5150	SNV	1	C	T	Homozygous	2507	3833	65.41	0.487	36.68967

Supplementary Table S3

Country	Sample collection year	Host Species	Clade	N gene (Genbank ID)	G gene (Genbank ID)	Subregion (if Georgia)
Afghanistan	2004	Dog	Arctic-related	NA	EU086128	
Afghanistan	1996	Dog	Arctic-related	EU086162	NA	
Azerbaijan	2002	Dog	Cosmopolitan	LN879480	LN879480	
Bosnia and Herzegovina	1986	Wolf	Cosmopolitan	U42704	NA	
Bosnia and Herzegovina	1986	Red fox	Cosmopolitan	U42706	NA	
Cambodia	1998; 1999	Dog	Asian	EU086167; EU086168; EU086169; EU086171	EU086130; EU086168; EU 086131; EU086132; EU086133	
China	1998	Dog	Asian	EU086173	EU086135	
China	1992	Human	Asian	EU086176	EU086139	
China	2005	Dog	Asian	EU086186	EU086146	
China	2013	Fallow deer	Asian	KJ564280	KJ564280	
China	1994	Dog	Asian	KX148264	KX148264	
Cote d'Ivoire	1992	Dog	Africa-2	KX148232	KX148232	
Gabon	1986	Dog	ND	NA	AF325470	
Georgia	2015	Dog	Cosmopolitan	MT079901; MT079924; MT079919	NA	Adjara
Georgia	2015	Dog	Cosmopolitan	MT079892; MT079903	NA	Guria
Georgia	2015	Dog	Cosmopolitan	MT079904; MT079891; MT079895	NA	Imereti
Georgia	2015	Dog	Cosmopolitan	MT079896; MT079928; MT079909	NA	Kakheti
Georgia	2015	Dog	Cosmopolitan	MT079889; MT079897; MT079927	NA	Kvemo Kartli
Georgia	2016	Golden jackal	Cosmopolitan	MW055109	NA	no data
Georgia	2015; 2016	Dog	Cosmopolitan	MT079906; MT079926; MT079900	NA	Samegrelo-Zemo Svaneti
Georgia	2015	Dog	Cosmopolitan	MT079910; MT079912; MT079913	NA	Shida Kartli
Georgia	2015	Jackal	Cosmopolitan	MT079888	NA	Tbilisi
Georgia	2015	Dog	Cosmopolitan	MT079893; MT079894; MT079905	NA	Tbilisi
Greenland	1980	Dog	Arctic-related	KX148105	KX148105	
Guinea	1986	Dog	ND	NA	AF325484	
Hungary	1991	Human	Cosmopolitan	U43025	AF325462	
India	2006	Human	Arctic-related	EF437215	EF437215	
India	ND	Dog	Indian subcontinent	AF374721		
Indonesia	2003	Dog	Asian	EU086192	EU086151	
Iran	ND	Jackal	Cosmopolitan	KX148212; JX987746	KX148212	
Iran	ND	Dog	Cosmopolitan	MK760760	MK760760	
Iran	1987	Wolf	Cosmopolitan	U22483	NA	
Kazakhstan	1988	Red fox	Cosmopolitan	AY352489	NA	
Laos	1999	Dog	Asian	EU086193	EU086152	
Morocco	1986	Dog	ND	NA	AF325467	
Myanmar	1999	Dog	Asian	EU086165	EU086129	

Nepal	1998	Dog	Indian subcontinent	EU086196	EU086153	
Nepal	2011	Cattle	Indian subcontinent	KX148108	KX148108	
Philippines	2004	Human	Asian	EU086205	EU086155	
Poland	1985	Raccoon dog	Cosmopolitan	U22840	AF325464	
Russia	ND	Dog	Cosmopolitan	JQ944706	JQ944706	
Russia	1996	Dog	Cosmopolitan	AY352481	NA	
South Africa	2013	Mongoose	Africa-3	KX148220	KX148220	
Tajikistan	2012	Dog	Cosmopolitan	KY765901	KY765901	
Thailand	ND	Dog	Asian	JN786877	JN786877	
Turkey	ND	Dog	Cosmopolitan	KY860610	KY860610	
USA	2005	Bat	Bat	JQ685902	JQ685902	
Vietnam	2001	Dog	Asian	EU086209	EU086159	
